# Analysis of the Membrane Proteome of Ciprofloxacin-Resistant Macrophages by Stable Isotope Labeling with Amino Acids in Cell Culture (SILAC)

**DOI:** 10.1371/journal.pone.0058285

**Published:** 2013-03-07

**Authors:** Nancy E. Caceres, Maarten Aerts, Béatrice Marquez, Marie-Paule Mingeot-Leclercq, Paul M. Tulkens, Bart Devreese, Françoise Van Bambeke

**Affiliations:** 1 Pharmacologie cellulaire et moléculaire, Louvain Drug Research Institute, Université catholique de Louvain, Brussels, Belgium; 2 Laboratorium voor Eiwitbiochemie en Biomoleculaire Engineering, Universiteit Gent, Belgium; University of Cambridge, United Kingdom

## Abstract

Overexpression of multidrug transporters is a well-established mechanism of resistance to chemotherapy, but other changes may be co-selected upon exposure to drugs that contribute to resistance. Using a model of J774 macrophages made resistant to the fluoroquinolone antibiotic ciprofloxacin and comparing it with the wild-type parent cell line, we performed a quantitative proteomic analysis using the stable isotope labeling with amino acids in cell culture technology coupled with liquid chromatography electrospray ionization Fourier transform tandem mass spectrometry (LC-ESI-FT-MS/MS) on 2 samples enriched in membrane proteins (fractions F1 and F2 collected from discontinuous sucrose gradient). Nine hundred proteins were identified with at least 3 unique peptides in these 2 pooled fractions among which 61 (F1) and 69 (F2) showed a significantly modified abundance among the 2 cell lines. The multidrug resistance associated protein Abcc4, known as the ciprofloxacin efflux transporter in these cells, was the most upregulated, together with Dnajc3, a protein encoded by a gene located downstream of *Abcc4.* The other modulated proteins are involved in transport functions, cell adhesion and cytoskeleton organization, immune response, signal transduction, and metabolism. This indicates that the antibiotic ciprofloxacin is able to trigger a pleiotropic adaptative response in macrophages that includes the overexpression of its efflux transporter.

## Introduction

The plasma membrane is a receptacle to key molecules vital for the cellular integrity, and critical activities such as self-recognition, environment sensing, and communication are mediated by membrane proteins. Substance transport across membranes is fundamental to the maintenance of cellular homeostasis and is accomplished by ATP-binding cassette (ABC) transporters, one of the most conserved family of proteins present in all cell types [1], [2]. Under environmental changes, cells respond by altering their gene expression to restore homeostasis and survive. This is well illustrated in cells exposed to potentially toxic drugs in which diverse cytoprotective mechanisms can be set off conferring to them a resistant phenotype. A commonly observed biological strategy is the reduction of the intracellular drug accumulation by overexpression of multidrug efflux transporters of the ABC superfamily [3]. Yet, drug-resistant cells may present other less evident mechanisms operating synergistically. The fact that drug-resistant cell lines present higher levels of resistance than their transfected counterparts suggests indeed that other uncharacterized functions might contribute to the resistance [3]. In this respect, profiling of protein abundance may help to uncover the extent of the impact of chronic exposure to a toxic drug.

Previous work from our laboratory has shown that the fluoroquinolone antibiotic ciprofloxacin can select for a resistant phenotype in J774 mouse macrophages that had been exposed chronically to high concentrations of this drug [4]. This phenotype is characterized by a reduced accumulation and an increased efflux of the drug that we could attribute to an increase in the expression of the multidrug transporter Mrp4 encoded by the *Abcc4* gene located on chromosome 14 [5]. Molecular cytogenetic experiments showed that this overexpression is linked to *Abcc4* gene overrepresentation, grading from a partial trisomy of chromosome 14 at the first step of selection, to low-level amplifications of *Abcc4* locus, up to high-level amplification of *Abcc4* as homogeneous staining region (hsr), inserted on 3 different derivative chromosomes [6].

To examine whether exposure and resistance to the toxic effects of ciprofloxacin could be associated with other, unanticipated changes in the membrane proteome, we have now performed a global quantitative analysis using stable isotope labeling with amino acids in cell culture (SILAC) technology to compare the proteins expressed in membranes isolated from ciprofloxacin-resistant (CIP-R) J774 murine macrophages vs. their wild-type (WT) counterparts. SILAC is a potent technique for direct qualitative as well as quantitative comparison of proteomes [7]. It has been successfully used to evidence biological pathways altered in cisplatin-resistant cells [8]. Here, we have focused our analysis on enriched membrane fractions isolated from sucrose gradient interfaces, which allowed us to confirm a large abundance of Mrp4 in CIP-R cells. Yet, we also evidenced a modulation in the expression of multiple surface and intracellular membrane proteins with variety of functions. The data therefore suggest that along with the acquisition of a targeted-resistant mechanism (Mrp4 overexpression), cells surviving the chronic stress imposed by high ciprofloxacin concentrations simultaneously modify multiple pro-survival pathways to reach a new homeostatic equilibrium. In a broader context, they demonstrate the interest of the SILAC approach for unraveling pleiotropic changes occurring in cells upon drug exposure.

## Results and Discussion

### Membrane Proteins Preparation

**Figure 1:**
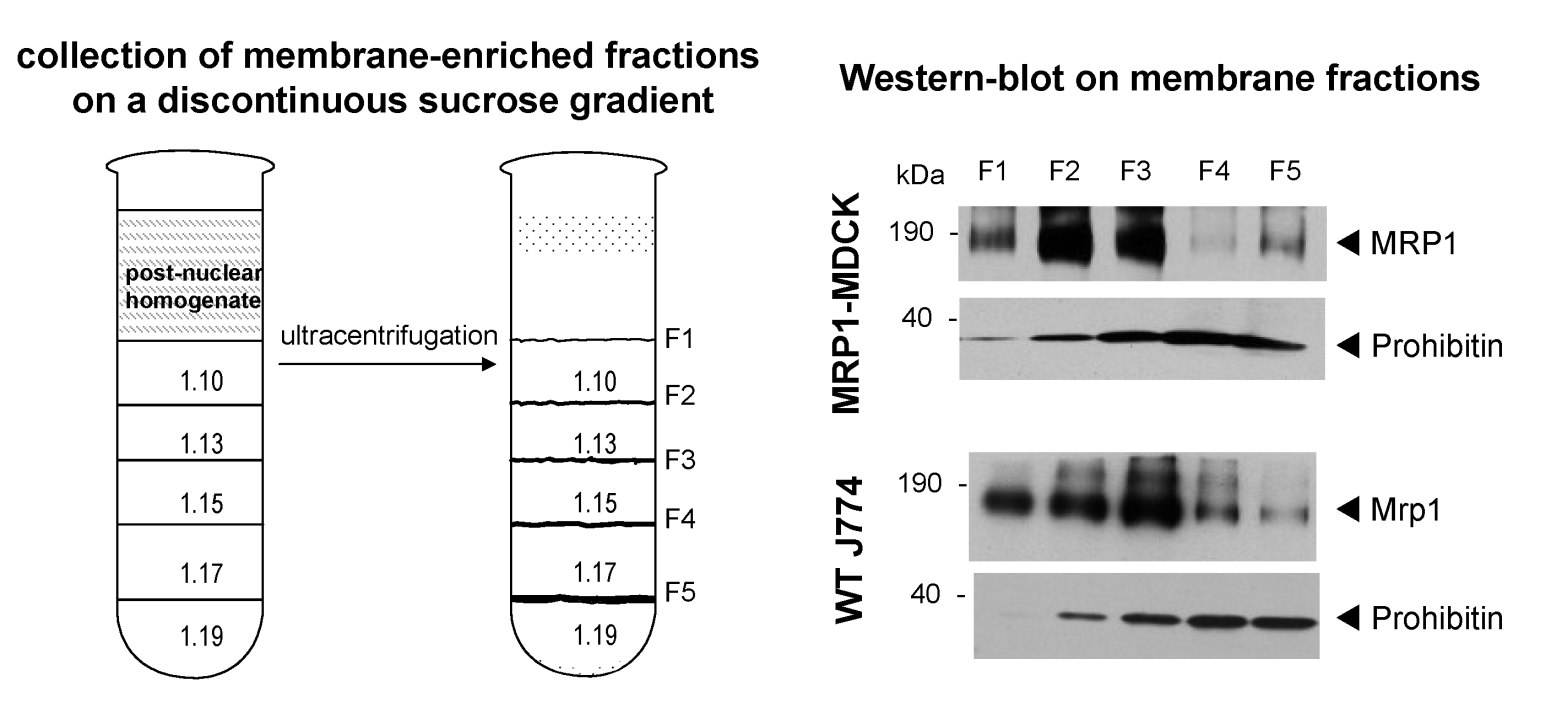
Plasma membrane enrichment strategy. Left: Illustration of the discontinuous sucrose gradient. Sucrose solutions densities (see materials and methods) and the resulting interphases (F1 to F5) are depicted before (thin lines) and after centrifugation (irregular lines). Right: Expression of a plasma membrane protein MRP1 and prohibitin, a mitochondrial marker in sucrose interphases. MRP1 and prohibitin were detected by Western blot in each of the interphases of MDCKII-MRP1 cells (top) and wild-type J774 macrophages (bottom). Anti-MRP1 (1∶2000) and anti-prohibitin (1∶1000) antibodies were followed by the appropriate anti-IgG HRP-labeled antibodies.

**Figure 2:**
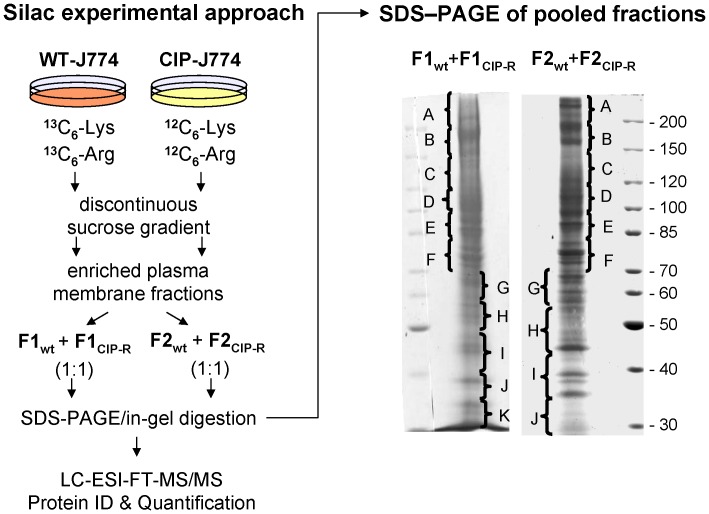
Overview of the SILAC experimental approach. Left: WT-J774 and CIP-R cells were cultured in media containing ^13^C_6_-Lys/^13^C_6_-Arg and ^12^C_6_-Lys/^12^C_6_-Arg, respectively. Following isolation of membrane samples for each strain independently, equal amounts from each matching fraction were pooled (F1_wt_+F1_CIP_ and F2_wt_+F2_CIP_) and then separated by SDS-PAGE. Right: Lanes of pooled sample fractions were first cut in sections (A-K for pooled F1 and A-J for pooled F2) and further sliced in thinner, numbered gel plugs which were processed for Trypsine in-gel digestion as described in the experimental procedures. For pooled F1 gel line, section A includes gel plugs from 1 to 8, B 9 to 14, C 15 to 20, C 21 to 24, E 25 to 29, F 33 to 35, G 36 to 41, H 42 to 45, I 46 to 52, J 53 to 60 and K 61 to 65. For sample pooled F2 gel line, section A includes bands 1 to 6, B 7 to 11, C 12 to 18, D 19 to 25, E 26 to 32, F 33 to 39, G 40 to 47, H 48 to 56, I 57 to 65 and J 66 to 72.

Figure 1 shows the isolation of samples enriched in plasma membrane by the discontinuous sucrose gradient method. The multipass plasma membrane transporter MRP1 was used as an indicator for enrichment in cell surface proteins and MDCKII-MRP1 cells overexpressing this protein were used to set up the fractionation conditions and the optimized protocol was applied to J774 macrophages. MRP1 levels were monitored by Western blotting of the fractions isolated from 5 sucrose density-interphases (F1 to F5). The mitochondrial marker prohibitin was used to evaluate the purity of each fraction, given that this organelle is the most common contaminant in this kind of preparations [9]. When using MDCKII-MRP1 cells, protein samples collected from interphases F1 to F3 showed increased MRP1 levels with a concomitant rise in prohibitin. Samples from WT-J774 macrophages presented an enrichment of Mrp1 in the equivalent fractions, with F1 and F2 displaying the lowest mitochondrial contamination. Based on these results, membrane samples isolated at the sucrose density-interphases F1 and F2 were selected for comparative and quantitative proteomic analysis, using pooled F1 (F1**_WT_**+F1**_CIP-R_**) and F2 (F2**_WT_**+F2**_CIP-R_**) membrane samples as depicted in Figure 2. A possible drawback in this experimental design is that membranes samples for each WT and CIP-R cells, had to be prepared and isolated independently (because of the intensive labor and instrumental constraints), and mixed only prior to the separation by SDS-PAGE. We can therefore not exclude slight variations in the purification conditions.

### Protein Identification by LC-MS/MS

After LC-MS analysis of the SDS-PAGE gel bands, a total of 122,542 and 111,580 MS/MS spectra were acquired from the pooled membrane fractions F1 and F2, respectively. For each pooled fraction, after protein database searching and automatic validation using TPP’s PeptideProphet and ProteinProphet tools, 45,947 and 49,268 peptides were identified, accounting for 1061 and 1144 proteins in pooled membrane fractions F1 and F2, respectively. At peptide level, PeptideProphet applied a 70% confidence threshold for positive identifications. During the further processing of the ProteinProphet results, EXCEL**®** exported protein lists of individual LC-MS experiments were reorganized using in-house written VBA-scripts. Only protein hits with probabilities higher than 0.95 were retained, and redundant hits, appearing in multiple, often adjacent gel bands, were grouped. No decoy proteins were identified with more than 2 unique peptide sequences. Therefore, a list of proteins with at least 3 unique peptides identified within an individual LC-experiment was generated reflecting very significant and unambiguously identified proteins (Table S1). A total of 651 and 735 proteins were unambiguously identified in the pooled F1 sample and pooled F2 sample, respectively, out of which 486 were present in both fractions, leading to a total number of 900 unique mouse proteins recognized. Additionally, 386 and 381 proteins represented by only 1 or 2 peptides were recovered in pooled fraction F1 and F2 with a respectively false discovery rate of 3.5% and 4.5%, correlating well with the 0.95 significance threshold used by ProteinProphet (Table S2).

**Figure 3:**
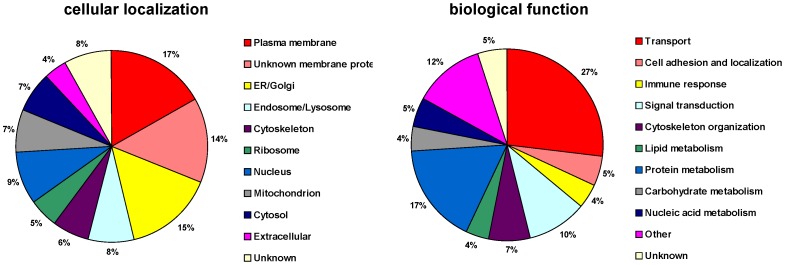
Classification of the identified proteins. Distribution charts of cellular localization (left) and biological function (right) of all proteins identified with high significance in both pooled F1 and F2 fractions. Proteins with multiple cellular localizations or biological functions are assigned to the one it is best known. Transporter proteins are involved in both transmembrane and intracellular transport.

To assess the contribution of proteins from plasma membrane and other cellular organelles to the total protein hits, an analysis according to the cellular compartment and biological function was performed based on the protein’s Gene Ontology (GO) terms. From all identified proteins, 17% were plasma membrane proteins. Proteins from the endomembrane system accounted for 44% of the sample proteins (15% from ER/Golgi, 5% from ribosomes, 7% from mitochondria, 8% from endosomes/lysosomes, and 9% from nucleus) whereas 17% were assigned as unknown membrane proteins. Finally, 6% of the sample corresponded to structural proteins of the cytoskeleton, 7% to cytosolic and 4% to extracellular proteins (Figure 3, left). Based on their biological function (Figure 3, right), most of these proteins are involved in transmembrane or intracellular transport mechanisms (27%) or metabolic processes (30%; among which 17% in the metabolism of proteins; 5%, of nucleic acids; 4%, of carbohydrates; and 4%, of lipids).

### Quantitative SILAC-based Analysis

**Figure 4:**
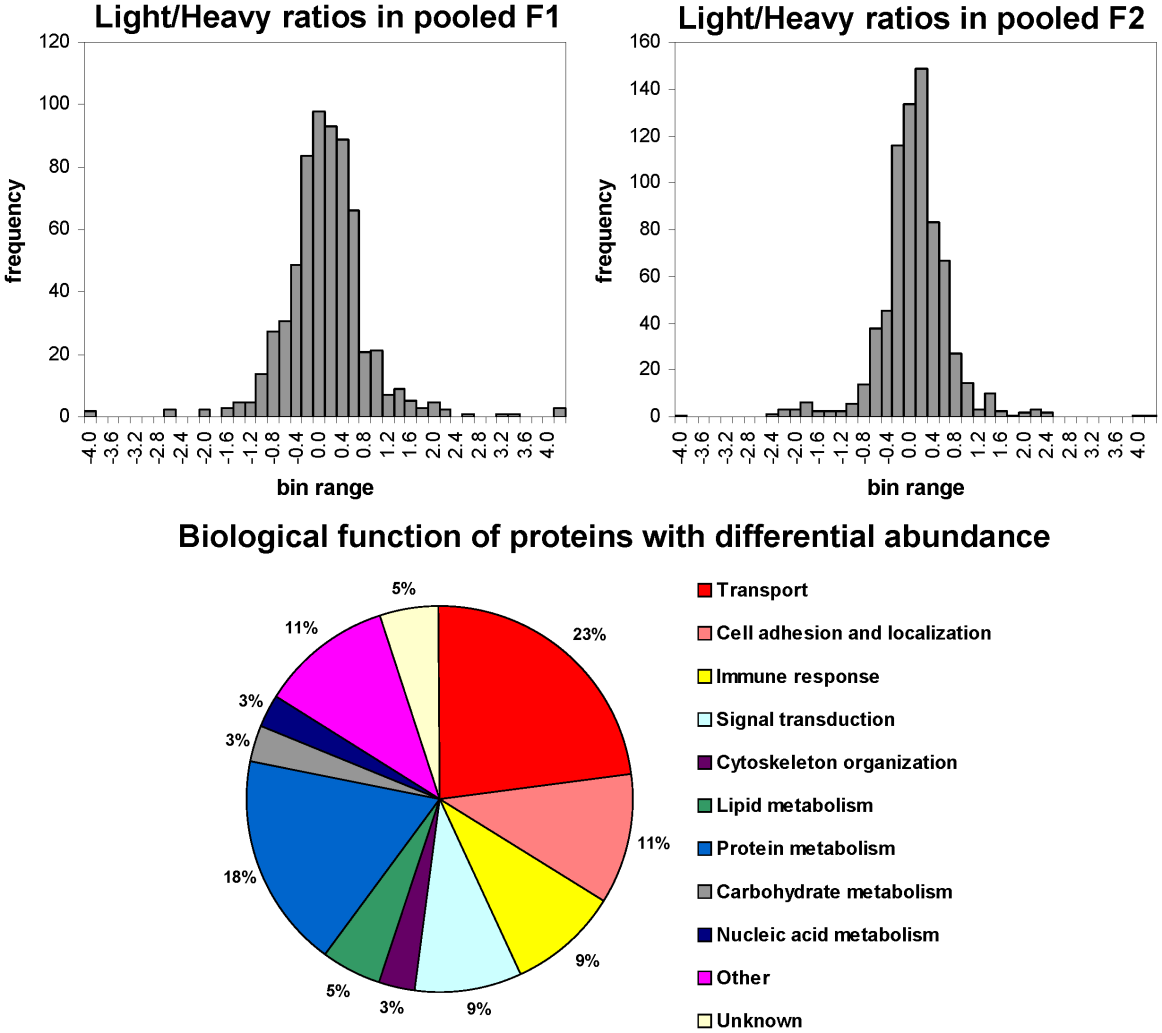
Sample quantization and classification of modulated proteins. Top: Histograms showing distribution of log_2_ protein Light/Heavy ratios after normalization toward the median for proteins identified by 3 or more tryptic peptides from pooled F1 and F2 samples, respectively. After normalization the distributions fitted to a Gaussian curve indicating that protein ratios have a median of 0 on the log2 scale as expected for a 1∶1 mixture. Bottom: Distribution chart of the biological function of proteins that show a 95% significant differential abundance ratio in pooled fraction F1 or F2.

Final protein abundance ratios were calculated by averaging the logarithmic based 2 transformed L/H ratios of a particular protein scattered in multiple, often adjacent, gel bands. After normalization towards the median, the resulting histograms fit a Gaussian distribution for both pooled membrane fractions (Figure 4). Therefore, albeit that pooling of light (CIP-R) and heavy (WT) labeled proteins was done after purification of the membrane fractions, the mixture was composed of a 1∶1 proportion, validating the experimental method and parameters. In pooled fraction F1, we detected 61 unambiguously identified proteins that show a significant (P-value <0.05) difference in protein abundance ratios between WT and CIP-R cells (Table 1). Similarly, pooled fraction F2 data demonstrated 69 proteins with altered protein abundance between both proteomes (Table 2). Several proteins display a significant change in expression level in only one of these two fractions. These differences are expected in our approach, where pooled F1 and F2 fractions differ in their enrichment in plasma membrane and constitute therefore partially different proteomes.

**Table 1:** Proteins with change in expression levels in pooled fractions F1.

#	Accession	Protein name	Log_2_L/H	P-value	Coverage	F2
Over-expressed proteins						
1	Q3TZN9	ABCC4/MRP4 efflux transporter	+ a	0.000	34.9	+ (0.00)
2	Q91YW3	DnaJ homolog subfamily C member 3 (p58IPK)	4.48	0.000	20.2	3.83(0.00)
3	Q3TYT8	Collectin-12	3.34	0.000	6.1	*−0.38(0.34)*
4	P01899	H-2 class I histocompatibility antigen, D-B alpha chain precursor	3.06	0.000	12.4	2.32(0.00)
5	Q3U651	Cathepsin D	2.44	0.000	5.1	ni.
6	P01900	H-2 class I histocompatibility antigen. D-D alpha chain precursor	2.16	0.000	37.5	1.80(0.00)
7	Q91VK4	Integral membrane protein 2C (CT-BRI3)	2.00	0.000	20.4	*0.73(0.24)* *
8	P28798	Granulins precursor (proepithelin)	1.99	0.000	13.9	ni.
9	Q810U4	Neuronal cell adhesion molecule precursor (NrCam)	1.95	0.000	13.6	2.28(0.00)
10	P10404	MLV-related proviral Env polyprotein precursor	1.90	0.000	6.2	*−*0.14(0.72)
11	Q61207	Sulfated glycoprotein 1 precursor (prosaposin)	1.81	0.001	11.3	1.33(0.00)
12	P12023	Amyloid beta A4 protein precursor (APP)	1.75	0.001	12.3	1.41(0.02)*
13	P97333	Neuropilin-1 precursor (CD304)	1.64	0.002	22.0	1.38(0.00)
14	O09126	Semaphorin-4D precursor (CD100)	1.63	0.002	8.7	ni.
15	Q80V26	Inositol monophosphatase 3	1.58	0.003	6.2	1.77(0.00)*
16	P06797	Cathepsin L1 precursor	1.56	0.004	34.4	*0.44(0.31)* *
17	Q61469	Lipid phosphate phosphohydrolase 1 (PAP-2a)	1.53	0.004	5.7	ni.
18	Q8K482	EMILIN-2 precursor	1.48	0.006	8.3	*0.42(0.33)*
19	P03975	IgE-binding protein (IAP)	1.48	0.006	19.4	1.99(0.00)
20	Q9D620	Rab11 family-interacting protein 1 (Rab-coupling protein)	1.33	0.013	7.6	1.31(0.00)
21	P42082	T-lymphocyte activation antigen CD86	1.32	0.014	20.4	0.87(0.04)
22	Q60751	Insulin-like growth factor 1 receptor precursor (CD221)	1.29	0.016	8.3	ni.
23	Q05910	ADAM 8 precursor (CD156a)	1.28	0.017	11.6	1.92(0.00)
24	Q8BWW9	Serine/threonine-protein kinase N2	1.27	0.017	14.3	ni.
25	P01902	H-2 class I histocompatibility antigen, K-D alpha chain precursor	1.25	0.019	29.3	0.99(0.02)
26	P27512	Tumor necrosis factor receptor superfamily member 5 precursor (CD40)	1.21	0.023	12.8	*0.73(0.24)* *
27	P10923	Osteopontin precursor	1.21	0.024	15.0	*−0.66(0.10)*
28	P16675	Lysosomal protective protein precursor (Cathepsin A)	1.20	0.025	14.1	ni.
29	A2AR26	Solute carrier family 2, member 6 (Slc2a6)	1.18	0.027	7.9	2.09(0.00)
30	O89103	Complement component C1q receptor precursor	1.18	0.028	30.0	*0.57(0.19)*
31	O89017	Legumain precursor	1.16	0.030	17.7	*0.47(0.45)* *
32	O35474	EGF-like repeat and discoidin I-like domain-containing protein 3 precursor	1.14	0.033	9.6	*−0.90(0.02)*
33	P35951	Low-density lipoprotein receptor precursor	1.12	0.036	12.0	*0.82(0.06)*
Lower-expressed proteins						
1	P58681	Toll-like receptor 7 precursor	*−*4.41	0.000	11.0	*−*3.89(0.00)
2	P47738	Aldehyde dehydrogenase, mitochondrial precursor	*−*2.74	0.000	24.1	*−*1.41(0.00)
3	P53026	60S ribosomal protein L10a	*−*2.61	0.000	18.9	*0.13(0.76)*
4	A1L314	Macrophage expressed gene 1 protein (Mpeg1)	*−*2.14	0.000	18.0	*−*1.77(0.00)
5	Q03265	ATP synthase subunit alpha, mitochondrial precursor	*−*2.07	0.000	28.2	*−*0.92(0.02)
6	Q61549	Cell surface glycoprotein F4/80	*−*1.78	0.001	6.8	ni.
7	P24063	Integrin alpha-L precursor (CD11a)	*−*1.72	0.002	21.8	*−*2.37(0.00)
8	Q3TVQ0	Formyltetrahydrofolate synthetase domain containing 1	*−*1.61	0.003	4.1	*−0.77(0.06)*
9	Q7TPV4	Myb-binding protein 1A	*−*1.54	0.004	11.0	ni.
10	Q8CGK3	Lon protease homolog, mitochondrial precursor	*−*1.54	0.004	4.0	ni.
11	P26443	Glutamate dehydrogenase 1, mitochondrial precursor	*−*1.42	0.009	9.5	*−0.53(0.18)*
12	Q3TFD0	Serine hydroxymethyltransferase	*−*1.42	0.009	22.8	*−0.53(0.18)*
13	P51675	C-C chemokine receptor type 1 (CD191)	*−*1.42	0.009	8.2	ni.
14	P63038	60 kDa heat shock protein, mitochondrial precursor	*−*1.37	0.012	36.8	*−0.50(0.21)*
15	Q99KI0	Aconitate hydratase, mitochondrial precursor	*−*1.37	0.012	11.2	*−0.38(0.34)*
16	P38647	Stress-70 protein, mitochondrial precursor	*−*1.37	0.012	8.0	*−0.10(0.79)*
17	Q6P5F7	Protein tweety homolog 3	*−*1.37	0.012	4.8	ni.
18	Q8CGC6	RNA-binding protein 28	*−*1.26	0.020	4.0	ni.
19	Q91VE6	MKI67 FHA domain-interacting nucleolar phosphoprotein	*−*1.16	0.033	13.2	*−0.05(0.90)*
20	P20152	Vimentin	*−*1.13	0.038	67.4	*−*1.94(0.00)
21	Q69ZN7	Myoferlin	*−*1,12	0.039	54.2	*−*1.76(0.00)
22	P21956	Lactadherin precursor	*−*1,11	0.040	21.8	*−0.57(0.15)*
23	P57746	Vacuolar ATP synthase subunit D	*−*1,11	0.040	18.6	*−*1.49(0.00)
24	Q60932	Voltage-dependent anion-selective channel protein 1	*−*1,09	0.045	39.9	*−0.69(0.15)* *
25	Q3TJG0	Band 4.1-like protein 5	*−*1,07	0.049	8.1	ni.
26	P50516	Vacuolar ATP synthase catalytic subunit A	*−*1,07	0.049	42.5	*−*1.92(0.00)
27	Q9DBG7	Signal recognition particle receptor subunit alpha (SRPα)	*−*1,07	0.049	8.5	ni.
28	Q8JZR0	Long-chain-fatty-acid–CoA ligase 5	*−*1,07	0.049	4.1	*−0.38(0.33)*

List of the 61 differentially expressed proteins identified in pooled fractions F1, using a 0.95 significance threshold (P-value). Accession corresponds to the Uniprot accession number. Log_2_L/H gives the logarithm of the average ratio of light (CIP-R) labeled peptides over heavy (WT) labeled peptides to the base 2:+indicates that no heavy labeled peptides could be detected. Coverage is the sequence coverage percentile of identified peptides in each protein. F2 shows Log_2_L/H ratio and P-value of the protein in pooled fractions F2: values in italic do not meet the 0.95 significance threshold,

* protein identification and quantification is only based on 1 or 2 tryptic peptides, ni., not identified.

a signal in the WT cells not above the noise level.

**Table 2:** Proteins with change in expression levels in pooled fractions F2.

#	Accession	Protein name	Log_2_L/H	P-value	Coverage	F1
Over-expressed proteins						
1	Q3TZN9	ABCC4/MRP4 efflux transporter	+^a^	0.000	26.9	+(0.00)
2	Q91YW3	DnaJ homolog subfamily C member 3 (p58IPK)	3.83	0.000	20.0	4.48(0.00)
3	P01899	H-2 class I histocompatibility antigen, D-B alpha chain precursor	2.32	0.000	12.4	3.06(0.00)
4	Q810U4	Neuronal cell adhesion molecule precursor (NrCam)	2.28	0.000	7.1	1.95(0.00)
5	A2AR26	Solute carrier family 2, member 6 (Slc2a6)	2.09	0.000	11.7	1.18(0.03)
6	P63085	Mitogen-activated protein kinase 1 (p42-MAPK)	2.09	0.000	30.2	*−0.04(0.94)*
7	Q61213	Gag	2.02	0.000	4.6	1.84(0.03)*
8	P03975	IgE-binding protein (IAP)	1.99	0.000	36.6	1.48(0.01
9	P01900	H-2 class I histocompatibility antigen, D-D alpha chain precursor	1.80	0.000	23.3	2.16(0.00)
10	Q3UT74	Integrin alpha 9 protein (Itga9)	1.67	0.000	8.4	1.78(0.03)*
11	P26955	Cytokine receptor common subunit beta (CD131)	1.48	0.001	8.4	2.47(0.00)*
12	P97333	Neuropilin-1 precursor	1.38	0.001	15.0	1.64(0.00)
13	Q921H8	3-ketoacyl-CoA thiolase A, peroxisomal precursor	1.38	0.001	16.0	*0.75(0.16)*
14	Q61207	Sulfated glycoprotein 1 precursor (prosaposin)	1.33	0.002	7.5	1.81(0.00)
15	Q8C166	Copine-1	1.32	0.002	6.3	*0.62(0.25)*
16	Q9JLZ8	Single Ig IL-1-related receptor	1.31	0.002	7.8	*1.16(0.17)* *
17	Q9D620	Rab11 family-interacting protein 1 (Rab-Coupling protein)	1.31	0.002	24.5	1.33(0.01)
18	Q05910	ADAM 8 precursor (CD156a)	1.29	0.003	7.7	1.28(0.02)
19	Q99LS5	Tex264 protein	1.28	0.003	4.5	ni.
20	P12265	Beta-glucuronidase precursor	1.22	0.005	21.0	*0.78(0.14)*
21	Q3TYD6	Serine/threonine-protein kinase LMTK2	1.13	0.009	8.2	*0.88(0.29)* *
22	O54782	Epididymis-specific alpha-mannosidase precursor	1.08	0.012	7.1	*0.90(0.09)*
23	Q9DC29	Mitochondrial ATP-binding cassette sub-family B member 6	1.06	0.013	14.8	*1.04(0.05)*
24	Q9D1R9	60S ribosomal protein L34	1.05	0.015	23.1	ni.
25	P01902	H-2 class I histocompatibility antigen, K-D alpha chain precursor	0.99	0.021	26.4	1.25(0.02)
26	P12970	60S ribosomal protein L7a	0.98	0.023	33.1	*−0.82(0.13)*
27	Q09200	Beta-1,4 N-acetylgalactosaminyltransferase 1	0.98	0.023	20.1	*0.86(0.11)*
28	Q924S8	Sprouty-related, EVH1 domain-containing protein 1	0.97	0.024	11.3	*1.15(0.08)* *
29	P31996	Macrosialin precursor (CD68)	0.97	0.025	5.5	*0.50(0.35)*
30	P35979	60S ribosomal protein L12	0.92	0.033	15.2	ni.
31	Q61033	Lamina-associated polypeptide 2 (Lap2)	0.88	0.041	4.2	ni.
32	P42082	T-lymphocyte activation antigen CD86	0.87	0.044	22.3	1.32(0.01)
33	Q61735	Leukocyte surface antigen CD47	0.87	0.044	8.9	*0.92(0.08)*
34	P62270	40S ribosomal protein S18	0.85	0.048	17.1	ni.
Lower-expressed proteins						
1	P58681	Toll-like receptor 7 precursor	*−*3.89	0.000	15.0	*−*4.41(0.00)
2	P24063	Integrin alpha-L precursor (CD11a)	*−*2.37	0.000	8.1	*−*1.72(0.00)
3	Q3UQJ7	DnaJc 13 homolog, N-terminal fragment RME-8	*−*2.24	0.000	13.1	ni.
4	Q8BNL1	DnaJc 13 homolog, C-terminal fragment RME-8	*−*2.07	0.000	6.0	ni.
5	P62814	Vacuolar ATP synthase subunit B, brain isoform	*−*2.00	0.000	35.4	*−0.85(0.12)*
6	Q8BVE3	Vacuolar ATP synthase subunit H	*−*1.98	0.000	25.3	*−0.67(0.21)*
7	P20152	Vimentin	*−*1.94	0.000	30.0	*−*1.13(0.04)
8	P50516	Vacuolar ATP synthase catalytic subunit A	*−*1.92	0.000	48.8	*−*1.07(0.05)
9	P50518	Vacuolar ATP synthase subunit E 1	*−*1.91	0.000	34.5	*−0.90(0.20*)
10	Q9Z1G3	Vacuolar ATP synthase subunit C 1	*−*1.90	0.000	24.1	*−0.69(0.20)*
11	A1L314	Macrophage expressed protein (Mpeg1)	*−*1.77	0.000	8.4	*−*2.14(0.00)
12	Q69ZN7	Myoferlin	*−*1.76	0.000	32.6	*−*1.12(0.04)
13	P57746	Vacuolar ATP synthase pump subunit D	*−*1.49	0.000	29.6	*−*1.11(0.04)
14	P47738	Aldehyde dehydrogenase, mitochondrial precursor	*−*1.41	0.000	37.0	*−*2.74(0.00)
15	O89001	Carboxypeptidase D precursor	*−*1.34	0.001	5.3	*−0.67(0.21)*
16	Q3U829	Uncharacterized protein	*−*1.29	0.001	2.5	ni.
17	Q8R4Y4	Stabilin-1 precursor	*−*1.12	0.005	1.8	ni.
18	P70290	55 kDa erythrocyte membrane protein (p55)	*−*1.12	0.005	10.1	*−1.02(0.06)*
19	Q80VQ0	Aldehyde dehydrogenase 3B1	*−*1.11	0.005	22.6	*−0.92(0.09)*
20	Q7TPR4	Alpha-actinin-1	*−*1.06	0.008	26.9	*−0.72(0.21)* *
21	P17439	Glucosylceramidase precursor	*−*1.05	0.008	15.1	*0.19(0.73)*
22	Q9CPN8	Insulin-like growth factor 2 mRNA-binding protein 3	*−*0.96	0.015	26.4	*−0.53(0.37)* *
23	Q99K70	Ras-related GTP-binding protein C	*−*0.93	0.019	8.8	*−0.99(0.09)* *
24	P30204	Macrophage scavenger receptor types I and II (CD204)	*−*0.92	0.020	32.1	*−0.23(0.67)*
25	Q03265	ATP synthase subunit alpha, mitochondrial precursor	*−*0.92	0.020	32.7	*−*2.07(0.00)
26	P60122	RuvB-like 1	*−*0.92	0.020	9.0	ni.
27	O35474	EGF-like repeat and discoidin I-like domain-containing protein 3 precursor	*−*0.90	0.023	5.8	1.14(0.03)
28	Q91WB7	Ubiquitin domain-containing protein 1	*−*0.90	0.023	10.6	*−0.59(0.32)* *
29	P55302	Alpha-2-macroglobulin receptor-associated protein precursor	*−*0.87	0.028	9.7	ni.
30	Q91VR2	ATP synthase subunit gamma, mitochondrial precursor	*−*0.84	0.034	8.4	ni.
31	Q8BFR5	Elongation factor Tu, mitochondrial precursor	*−*0.82	0.038	5.8	ni.
32	Q8BIJ7	RUN and FYVE domain-containing protein 1 (Rabip4)	*−*0.82	0.040	13.3	*−0.44(0.46)* *
33	Q3UH76	plexin B2	*−*0.80	0.043	17.8	*−0.44(0.42)*
34	O08599	Syntaxin-binding protein 1	*−*0.80	0.043	9.9	*−0.11(0.83)*
35	Q9WV54	Acid ceramidase precursor	*−*0.79	0.046	18.3	*−0.10(0.85)*

List of the 69 differentially expressed proteins identified in pooled fractions F2, using a 0.95 significancy threshold (P-value). Accession corresponds to the Uniprot accession number. Log_2_L/H gives the logarithm of the average ratio of light (CIP-R) labeled peptides over heavy (WT) labeled peptides to the base 2:+indicates that no heavy labeled peptides could be detected. Coverage is the sequence coverage percentile of identified peptides in each protein. F1 shows Log_2_L/H ratio and P-value of the protein in pooled fractions F1: values in italic do not meet the 0.95 significance threshold,

* protein identification and quantification is only based on 1 or 2 tryptic peptides, ni., not identified.

a signal in the WT cells not above the noise level.

When examining the function of the differentially expressed proteins (Figure 4), we observed a small enrichment of proteins involved in cell adhesion, motility and immune response (11%, *vs.* 5% of identified proteins) in comparison with the totality of proteins identified. In contrast, proteins involved in structural functions were modulated in lesser extent (3%, vs. 7% of identified proteins associated with cytoskeleton).

### Proteins Modulated Upon Chronic Exposure to Ciprofloxacin

#### Overexpression of the multidrug transporter Abcc4 involved in ciprofloxacin efflux

**Figure 5:**
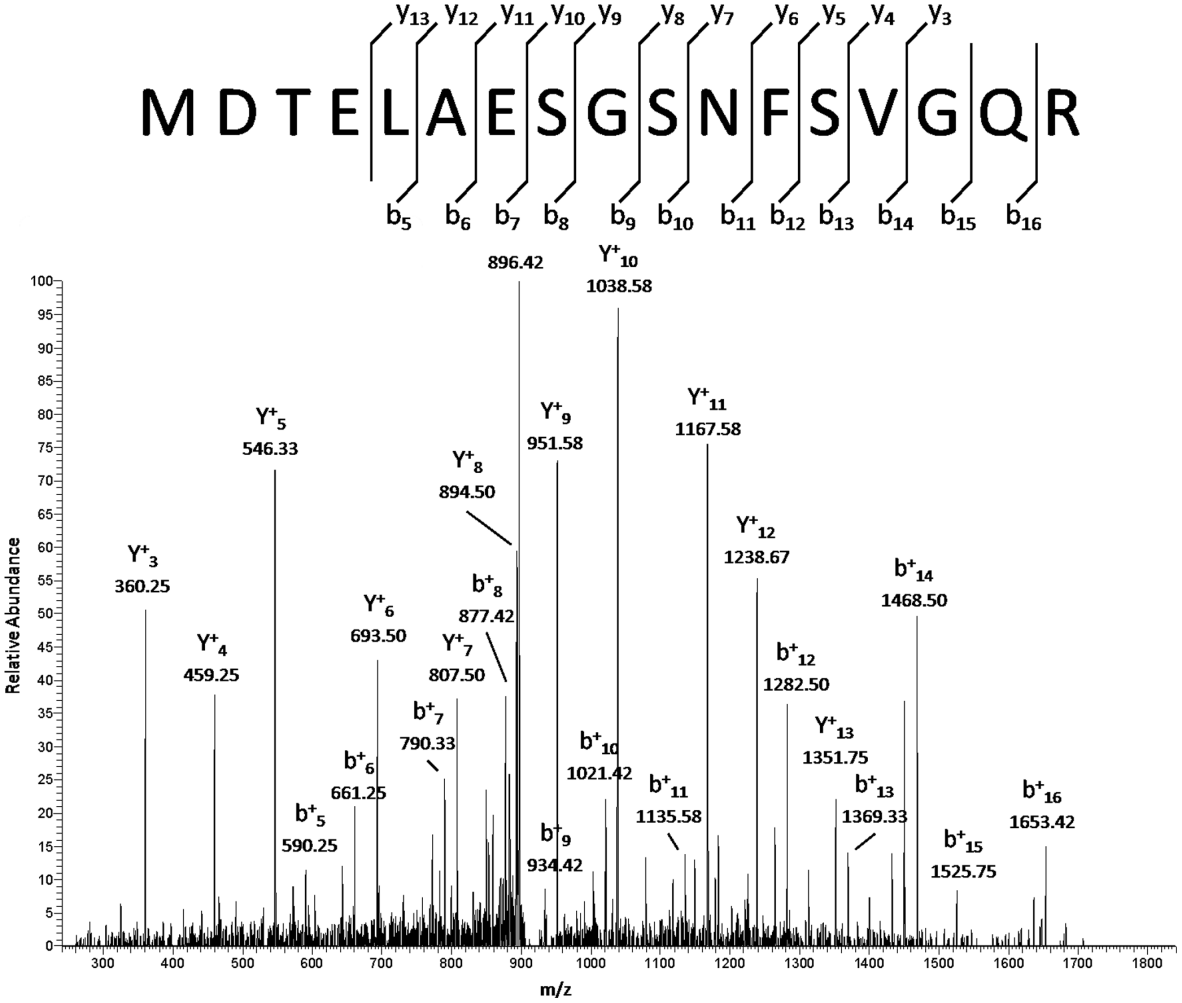
MS/MS spectrum of one of the Mrp4 peptides. The MS/MS spectrum is showing a CID fragmentation profile which was measured in the LTQ ion trap mass analyzer. The double charged precursor ion (m/z 914.4136) was previously detected by the high-resolution FT-ICR mass analyzer. Matched y- and b-ions are indicated above and below the amino acid sequence given in the one letter code.

In both membrane fractions, the multidrug transporter Abcc4/Mrp4 (Q3TZN9) belonging to the ABC superfamily [10] was the most abundant protein in CIP-R cells (see Figure 5 for an example of a MS/MS spectrum which contributed to the identification of Mrp4). This observation is coherent with our previous observation that Mrp4 is the main ciprofloxacin transporter in these cells and is markedly overexpressed at both the protein and mRNA levels in CIP-R cells [5]. As we were interested in multidrug resistance, we also looked for other ABC multidrug transporters. Only Abcb1b (Mdr1) (one of the two murine P-gp isoforms), and Abcc1 were detected, but without any difference of expression between the two cell lines (See Tables S1 and S2). Abcc2 (Mrp2), for which we have already demonstrated an overexpression in resistant cells, was not detected here, probably due to its low abundance [5]. Mrp4 has a natural function in the export of cyclic nucleotides like cAMP and proinflammatory mediator leukotriene [11], [12]. Massive production of Mrp4 may therefore have an impact on intra- en extracellular signaling as will be demonstrated below.

#### Overexpression of p58*^IPK^*/Dnajc3 and evaluation of resistance to ER stress

**Figure 6:**
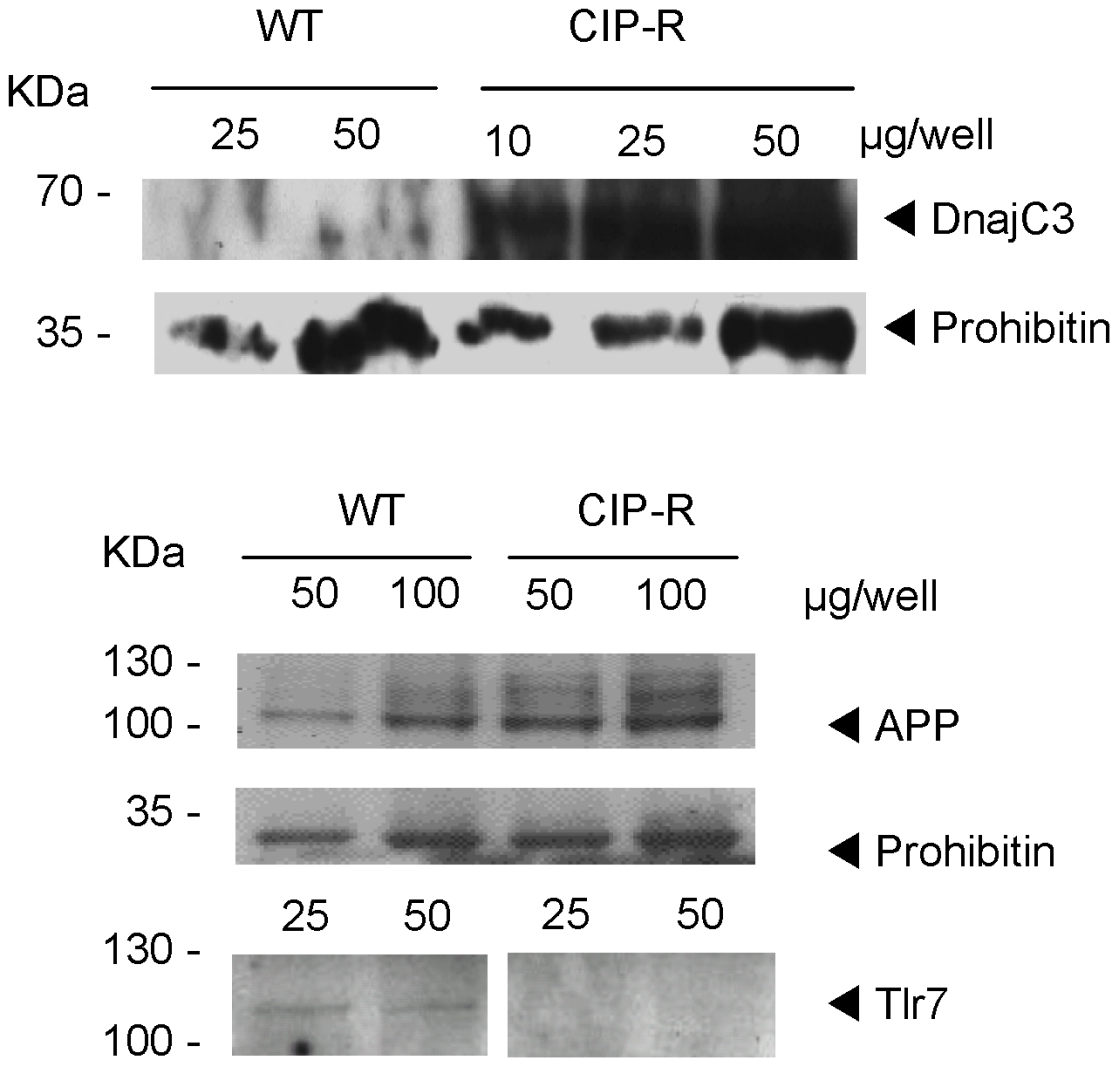
Validation by Western blot of differentially expressed proteins. Western blots of membrane proteins prepared from wild-type (WT) and ciprofloxacin-resistant (CIP-R) J774 macrophages. Gels were loaded with the indicated amounts of proteins. Top: Detection of Dnajc3 with a rabbit anti-mouse Dnajc3 polyclonal antiserum (1∶500) and prohibitin with an anti-prohibitin polyclonal antibody (1∶500) followed by the appropriate anti-IgG HRP-labeled antibodies. Bottom: Revelation of amyloid precursor protein App with a rabbit antiserum (1∶2000) and of prohibitin as loading control, followed by the appropriate anti-IgG HRP-labeled antibodies (1∶5000). For Tlr-7 detection, gels were loaded with the amounts of protein indicated and revealed with anti-Tlr7 antibody (1∶75), followed by the appropriate anti-IgG HRP-labeled antibody (1∶250).

**Figure 7:**
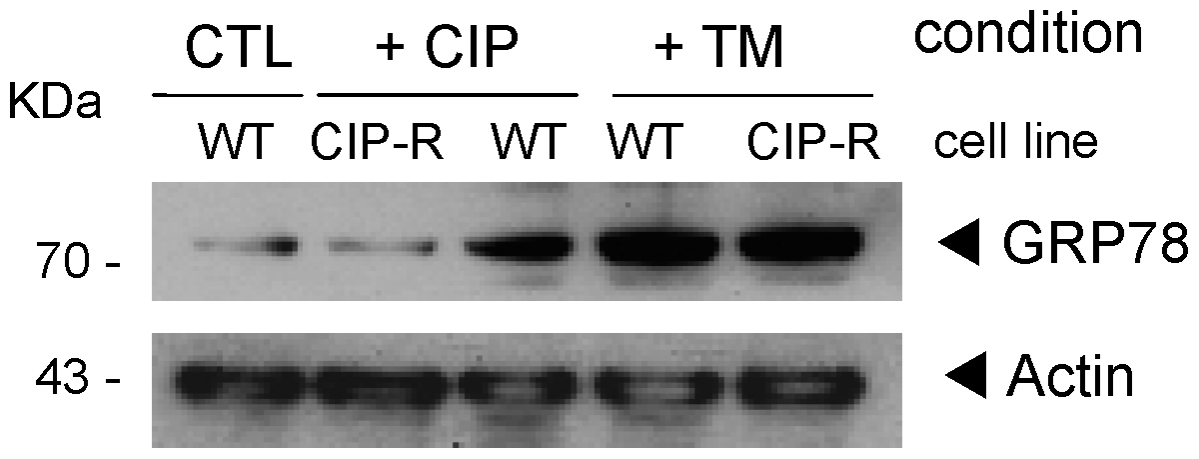
ER stress response evaluation. Immunoblot of Grp78 and actin as loading control in whole cell lysates from cells growing in standard conditions (line 1, WT cells in control medium; line 2, CIP-R cells in medium added by 0.2 mM ciprofloxacin), or in WT cells exposed for 24 h to 0.2 mM ciprofloxacin (line 3), or in cells exposed for 24 h to 0.5 µg/ml tunicamycin (TM; line 4, WT cells; line 5, CIP-R cells). The anti-Grp78 is a rabbit antiserum used at 1∶500 dilution. The hatched square highlights the standard conditions of culture for each cell line.

In both membrane fractions, P58***^IPK^***/Dnajc3 (Q91YW3) is also strongly more abundant in CIP-R macrophages. This was confirmed by Western-Blot (Figure 6). The corresponding gene is found 250 kB downstream of the *Abcc4* gene, located on mouse chromosome 14E4, suggesting that overproduction of the corresponding proteins is due to gene amplification of a chromosomal fragment. This hypothesis has been recently confirmed by FISH experiments demonstrating a co-amplication of these two genes [6]. P58***^IPK^***/Dnajc3 is a member of the DnaJ/Hsp40 (heat shock protein 40) co-chaperones family [13] and is considered to be an ER stress inducible chaperone and attenuator of the unfolding protein response (UPR [14], [15]). The massive expression of Mrp4 at the plasma membrane might generate a burden to the ER where membrane protein folding and maturation takes place. We therefore compared the response to ER stress in the two cell lines by measuring the expression of the ER chaperone Bip/Grp78 also known as Hspa5 heat shock protein 5 [16] in control conditions or after 24 h exposure to 0.2 mM ciprofloxacin or 0.5 µg/ml of tunicamycin, an UPR-stress inducer. Our SILAC analysis did not reveal any change in the expression of this protein (see Table S1) in standard conditions of culture (i.e. control medium for WT cells and medium added by 0.2 mM of ciprofloxacin for CIP-R cells), which was further confirmed by immunoblotting of whole cell lysates (Figure 6) or of membrane protein samples (not shown) from both cell lines. Yet, both ciprofloxacin and tunicamycin triggered a sustained over-expression of Grp78 in WT cells, but only tunicamycin in CIP-R cells (Figure 7). The absence of effect of ciprofloxacin in CIP-R cells could easily be attributed to the reduced cellular accumulation of the drug resulting from the overexpression of its efflux transporter [4]. Yet, the absence of protection against tunicamycin-induced stress in CIP-R cells rather suggests that the overexpression of the two proteins, Mrp4 and Dnajc3, is coincidental and related to their close location on the chromosome [6]. Most likely, P58***^IPK^***/Dnajc3 does not play a particular role in the resistant phenotype. This needs however to be further explored by examining other proteins involved in the ER stress cascade.

Moreover, P58***^IPK^***/DNAJC3 protects cells against apoptosis by preventing the activation of NF-κB signal cascades [17]. Of interest, ciprofloxacin has been shown capable of causing cell death by apoptosis in specific models (see [18] for a recent example). Intriguingly, however, some proteins that are under the transcriptional control of NF-κB are found more abundant in CIP-R cells, like MHC2-class I antigens [P01899, P01900, P01901, P01902] [19], neuronal cell adhesion molecule [Q810U4] [20], cathepsin L1 [P06797] [21], insulin-like growth factor1 receptor [Q60751] [22], CD40 [P27512], CD86 [P42082] [23], and osteopontin precursor [P10923] [24] but the NF-κB repressor Myb-binding protein 1A [Q7TPV4] [25] was down regulated.

#### Modulation of lipid synthesis

One of the physiological functions attributed to Mrp4 is the transport of lipid mediators like leukotrienes and prostaglandins [11], [26]. In this context, our analysis also highlights changes in the expression of proteins involved in lipid metabolism, which could have significant impact in the composition of the membranes and important consequences for intracellular transduction activities. In particular, the lipid phosphate phosphatase 1 (LPP-1or PAP-2a [Q61469]) and the inositol monophosphatase 3 [Q80V26] [27], [28], 2 proteins participating in the regulation of the activity of bioactive lipids and mediators of the intracellular phosphoinositide-based signaling [29], were upregulated. The low-density receptor protein LDRL [P35951] was upregulated as well [30]. On the contrary, two lysosomal hydrolases acting on lipids, namely glucosylceramidase [P17439] [31] and acid ceramidase [Q9WV54] [32] as well as the glycosyltransferase beta-1,4 N-acetylgalactosaminyltransferase 1 (GalNAc-T; Q09200) involved in the biosynthesis of gangliosides GM2, GD2 and GA2 [33] were downregulated in CIP-R cells. Some of these changes could be attributed to reduced cAMP levels, as for example for acid ceramidase [34].

#### Modulation of intracellular protein transport and recycling

Driving increased amounts of Mrp4 at the cell surface may require modulation of the intracellular pathways of protein transport and/or recycling. Although not optimized to detect proteins associated to intracellular membranes, our proteomic analysis detected changes in the expression of proteins implicated in intracellular trafficking and recycling in CIP-R macrophages. We found RAB11 family interacting protein 1 class 1 (Rab11-FIP1; Q9D620) positively modulated. This protein had been proposed to regulate Rab11 localization and to recruit additional factors to different endocytic compartments [35]. In contrast, the Rab4-interacting protein (RabIP4; Q8BIJ7), a protein that in conjunction with Rab4 seems to be involved in early endosomal traffic [36] was found down regulated in CIP-R cells. Apparently, RabIP4 could provide directionality to a backward traffic from the recycling endosomes to sorting endosomes [37]. The upregulated serine/threonine kinase lemur tyrosine kinase 2 (LMTK2; Q3TYD6) is involved in the transition of membrane vesicles from early endosomes to recycling endosomes and interacts with the actin-based retrograde motor protein myosin VI [38] involved in the movement of those vesicles [39]. Two Dnajc13 homologues, different from p58IPK (RME-8 [Q8BNL1] and Q3UQJ), involved in endosomal trafficking [40], were less expressed in CIP-R cells. In cells knock-out for RME-8, the level of cargos, such as the epidermal growth factor receptor (EGFR), is decreased while receptors that primarily recycle to the plasma membrane remain unaffected [41]. Two other proteins with a role in membrane trafficking were also modulated in the resistant cells, namely copine [Q8C166] [42], [43] and myoferlin [Q69ZN7] [44]. The link between these changes and functionality of Mrp4 at the cell membrane remains however to be established.

#### Modulation of adhesion and motility

Several integrins or associated proteins were found modified in our proteomic experiment. Thus, intregrin αL [P24063] was upregulated in CIP-R cells, while α9 [Q3UT74] and the leukocyte surface antigen CD47 [Q61735] (45) were downregulated. In addition, various adhesion molecules implicated in tumorigenesis were found up-regulated in ciprofloxacin-resistant macrophages. These include neuropilin-1 [P97333], which is implicated in cell proliferation, survival, and migration and protects cancer cells from apoptosis [46], the cell adhesion molecule NrCAM [Q810U4] that promotes cell growth and motility [47], [48], the extracellular matrix component and tumor progression marker osteopontin [P10923] [49], and the metalloproteinase-disintegrin ADAM8 [Q05910] [50].

#### Alteration of lysosomal proteome

Although the samples were selected to be enriched in pericellular membrane proteins, we also detected proteins associated to organelle membranes. In particular, we found modifications in the expression of a series of lysosomal proteins. Besides the lysosomal hydrolases acting on lipids mentioned above, many other proteins were found overexpressed in CIP-R cells, mainly: (i) precursor peptides of cathepsin A [P16675], D [Q3U651] and L1 [P06797] that intervene not only in proteolysis but also in inflammatory processes and apoptosis [51] (ii) legumain [O89017], a member of the C13 family of cysteine proteases, also found highly upregulated in many murine and human tumors [52] and (iii) prosaposin [Q61207], the precursor of lysosomal saposins A, B, C and D that has been associated with cancer [53], [54] and is described as having growth, migration, and invasion promoting activities and also anti-apoptotic effects [55]–[57]. It is established that lysosomal enzymes are first translated as proenzymes that become proteolytically activated within the lysosomes. Our GeLC approach therefore is able to separately identify and quantify the proteins processing states. Focusing on cathepsin B (P10605) for example, our data indicate a significant up-regulation of the cathepsin B precursor (MW 37.3 kDa), whereas the processed enzyme (MW 27.5 kDa) shows no regulation between WT and ciprofloxacin resistant cells (Figure S1 and Table S3). Averaging the ratios for the different peptides, however, results in a clear but not significant up-regulation of cathepsin B. Similar results are obtained for cathepsins L1 and D. In addition, six out of the eight (A to H) peripheral V1 subunits of the V-type ATPase [58] were significantly downregulated in CIP-R. Of interest, we noted previously that the basal level of ATP was increased in CIP-R cells [4], possibly to insure the increased need in energy needed for Mrp4 activity.

#### Alteration of cell signaling

A series of receptors playing a role in macrophage differentiation or activation were expressed to higher levels in CIP-R cells. This is the case for (i) the cytokine receptor common subunit β (CSF2RB, CD131) which is a shared component of Type I cytokine receptors (IL 3, IL 5 and GM-CSF) [59], (ii) the α and β chains of tumor necrosis factor receptor (CD40; P27512), a member of the tumor necrosis factor receptor (TNF) receptor family, (iii) the insulin-like growth factor 1 receptor (IGR-1 [Q60751] [60], a survival factor of malignant cells [61], and (iv) single Ig IL-1 related receptor (Sigirr) or TIR8 [Q9JLZ8], which regulates cancer-related inflammation and autoimmunity [62].

On the contrary, the Toll-like receptor 7 (TLR 7; P58681) was the lower expressed protein in resistant cells, in both analyzed fractions (see also Figure 6 for validation by western-blot). This could be consecutive to the dowregulation of Sigirr, a negative regulator of the IL-1 receptor/TLR system [62]. In the context of this study, an interesting characteristic of this protein is that it recognizes nucleosides and nucleotides [63] as does Mrp4.

Among proteins involved in differentiation, we found an increased expression of the histocompatibility H-2 class I antigen chain precursors (P01902). Interestingly, a proteomics study reports on the induction of major histocompatibility complex (MHC) class I molecules in activated macrophages [64]. On the contrary, we evidenced a reduction in the expression of a F4/F80 receptor [Q61549], a specific macrophage cell-surface marker for murine macrophages [65] and of the precursor of the macrophage scavenger receptors (MSR) I and II [P30204, which are expressed during differentiation of monocytes into macrophages [66].

#### Modulation of other cell surface proteins

Among other surface proteins, we also detected in CIP-R cells a down-regulation of Mpeg-1 [A1L314], a macrophage porin-like protein [67], and an up-regulation of semaphorin-4D (CD100 [O09126]) [68], of collectin-12 [Q3TYT8], a scavenger receptor [69], and of the C–C chemokine receptor type 1 CD191 [P51675] [70]. Peculiar outcomes were the overexpression of the amyloid beta (A4) precursor protein (App [P12023]; validated by Western-Blot, see Figure 6), the MLV related proviral Env polyprotein precursor [P10404], and the IgE binding protein or intracisternal A-Particle (IAP [P03975]). The IAP are encoded by retrovirus-like mobile elements present in the mouse genome [71] where they can transpose and act as mutagenic agents [72]. For instance, it has been shown that P-gp overexpression in a murine leukemic tumor cell line (gene *Abcb1a*) is caused by gene amplification and also transcriptional activation due to the insertion of a IAP in the promoter region of the gene [73]. Whether this is also the mechanism having led to gene amplification in our model needs to be determined experimentally. We also detected in CIP-R cells an increased amount of p42-MAPK [P63085], a serine/threonine kinase that controls a wide range of cellular activities [74]. Yet, peptides were located in bands corresponding to a much higher molecular weight than that expected for the denatured p42-MAPK polypeptide (band 6, >150 kDa).

### Conclusion

This study is, to the best of our knowledge, the first one to perform a SILAC analysis on purified membrane fractions of eukaryotic cells, constituting a potentially useful technological advance that may have several applications in biology or pharmacology.

Thus, the analysis performed here clearly shows that exposure of J774 macrophages to high concentrations of ciprofloxacin to obtain cells that resist to drug concentrations that would kill parent cells, induces pleiotropic alterations in protein expression. This observation is in the line of recent proteomic studies of drug-resistant cell lines that all highlight impressive modifications in the profile of protein expression, most often related to a diversity of metabolic pathways [8], [75]–[77]. In the present case, the most obvious change to explain the resistance phenotype is the overexpression of the ciprofloxacin efflux transporter, a mechanism observed for many other cytotoxic drugs. Quite intriguingly, however, co-amplification of P58***^IPK^***/Dnajc3 does not seem to contribute to resistance. Many of the other differentially expressed cell surface receptors and cell surface signaling elements evidenced in CIP-R macrophages are suggestive of activated macrophages, or contribute to cell motility, adhesion, cytoskeleton, immune response, activation of proinflammatory cytokines, angiogenesis, differentiation, lipid metabolism, and cell proliferation. Thus, virtually all of the regulated surface proteins in CIP-R cells have been linked to malignancies and/or in a broader context, conditions promoting cell growth and motility. It remains nevertheless to be determined whether these changes are contributing to resistance or are simply secondary to alterations in regulatory pathways, for example as a consequence of altered cAMP homeostasis. This would represent a piece of work by itself for each group of modified proteins associated with a specific biological function. Yet, confirming the significance of our results, transcriptomic studies (including microarray analysis) of human lymphocytes exposed to supratherapeutic concentrations of ciprofloxacin similar to those used here, also revealed modulations in mRNA of proteins belonging to major gene programs (like signal transduction factors, cytokines, adhesion and apoptosis related molecules), which share many commonalities with our own data [78], [79].

In a broader context, the fact that many of the proteins altered in their expression upon exposure to ciprofloxacin were similar to those modified with other cytotoxic agents (including chemotherapeutic agents), indicates that they are part of a global adaptative response to xenobiotics.

## Materials and Methods

### Chemicals

Ciprofloxacin (98% purity) was kindly provided as standard for microbiological evaluation by Bayer HealthCare A.G., Leverkusen, Germany. Camptothecin, tunicamycin and the zwitterionic detergents ASB14 (3-[N,N-Dimethyl(3-myristoylaminopropyl) ammonio]propanesulfonate Amidosulfobetaine-14) and C7BzO (3-(4-Heptyl)phenyl-3-hydroxypropyl)dimethylammoniopropanesulfonate) were obtained from Sigma-Aldrich (St. Louis, MO).

### Cell Culture

J774 mouse macrophages (referred to as WT cells) were grown in RPMI-1640 medium (Invitrogen, Carlsbad, CA) supplemented with 10% fetal bovine serum in 5% CO**_2_** atmosphere, as described previously [80]. J774 macrophages resistant to ciprofloxacin (referred to as CIP-R cells) were obtained as described earlier [4]. In brief, wild-type cells were cultivated in the presence of increasing concentrations of ciprofloxacin (from 0.1 mM to 0.2 mM) for about 50 passages (approx. 8 months), after which cells were maintained in the presence of 0.2 mM ciprofloxacin. MDCKII-MRP1 cells [81] were received from Prof. P. Borst (The Netherlands Cancer Institute, Amsterdam, The Netherlands), and grown in DMEM medium supplemented with 10% fetal bovine serum in 5% CO**_2_** atmosphere.

### Metabolic Labeling with Stable Isotopes

Before performing the SILAC experiment, a time course of incorporation of labeled amino acids was performed to establish the minimum time required for complete labeling of proteins. Both WT- and CIP-R macrophages were grown in the double labeled [U-**^13^**C**_6_**] L-Lysine, [U-**^13^**C**_6_**] L-Arginine SILAC medium (+6 Da shift) in 20 cm**^2^** dishes seeded at 1×10**^5^**cells/cm**^2^** (RPMI-1640 SILAC**™** Protein Identification and Quantitation Media Kit, Invitrogen). Whole cell lysates were prepared at various time points in 10 mM Tris-HCl, pH 7.4 plus 0.1% SDS and random peptides were analyzed by MS to evaluate incorporation of the heavy amino acids. Two passages were sufficient for the WT-J774 cells to reach complete incorporation of the heavy isotopes. SILAC labeling was thus performed with WT cells, while CIP-R cells were grown in medium with light amino acids.

### Preparation of Enriched Plasma Membrane Fractions

Cell monolayers (10 Petri dishes of 165 cm**^2^**, seeded at approx. 1.75×10**^5^** cells/cm**^2^**) were rinsed with cold PBS then detached with policeman and collected in the same buffer by centrifugation at 280×g for 10 min at 4°C. Sets of 5 monolayers were pooled and resuspended in 5 mL of 10 mM Tris-HCl, pH 7.4 plus protease-inhibitor cocktail-EDTA free (one tablet/10 ml; F. Hoffmann-La Roche Ltd Diagnostics Division, Basel, Switzerland). Cell suspensions were transferred to a Dounce B homogenizer (glass/glass, tight pestle) and stroked 25 times. The homogenates were centrifuged at 600×g for 10 min at 4°C. The post-nuclear supernatants were layered on the top of discontinuous sucrose gradients designed to separate membranes from the other main cell constituents [82]. Sucrose solutions were prepared in 10 mM Tris-HCl, pH 7.4 plus 0.5 mM phenylmethanesulphonyl fluoride (PMSF) and stored overnight at 4°C. Sucrose step gradients were assembled on ice, layering cold solutions of sucrose using a syringe with long needle in the following order: 1.5 ml of 1.10 density solution, 1 ml of 1.13 density solution, 1 ml of 1.15 density solution, 2 ml of 1.17 density solution and 2 ml of 1.19 density solution. Gradients were ultracentrifuged in a Beckman SW40 rotor at 280.000×g for 2 h at 4°C. The resulting visible bands at each interphase (Fig. 1A) constituted 5 different membrane fractions (F1 to F5). The five fractions were collected on ice and diluted to 6 ml with cold 0.1 M Na**_2_**CO**_3_**, pH 11 and centrifuged in a Type 50 Ti rotor at 100,000×g for 30 min at 4°C. The membrane pellets were then resuspended in 10 mM Tris-HCl, pH 7.4 plus protease-inhibitor cocktail-EDTA free and aliquots were removed for protein content determination using the bicinchoninic acid protein assay (Pierce, Rockford, IL). Membrane samples were then flash-frozen in an ethanol-dry ice bath and stored at –80°C for downstream analysis.

### Western Blot

After heating for 10 min at 70°C, membrane protein samples were loaded on acrylamide gels (NuPAGE Bis-Tris 4–12% gels, Invitrogen). After migration, separated proteins were transferred onto nitrocellulose membranes and then blocked overnight at 4°C in TBS-T (20 mM Tris-HCl, 500 mM NaCl pH 7.5 and 0.05% Tween 20) with 5% milk. Blotted membranes were exposed to appropriate primary antibodies, namely rabbit polyclonal anti-TLR7 (IMG-581A; Imgenex, San Diego, CA), rat monoclonal antibody anti-MRP1 (MRPr1, Alexis Biochemicals, Lausen, Switzerland), and sc-28259 (Santa Cruz Biotechnology, Santa Cruz, CA) to detect prohibitin, or to antisera against mouse DnajC3 [83], against the amyloid precursor protein APP (A8717, Sigma), or a rabbit antiserum against GRP78 (PA1-014A; Enzo Life Sciences International, Inc., Plymouth Meeting, PA; kindly provided by D. Tyteca, Institute de Duve and Université catholique de Louvain, Brussels, Belgium), followed by appropriate horseradish peroxidase-coupled secondary antibody (see figure captions for dilutions). Blots were revealed by a chemiluminescence assay (SuperSignal West Pico, Pierce).

### SDS-PAGE and In-Gel Digestion

Membrane fractions F1 and F2 were separately collected for each cell line (Fig. 2A). To extract proteins from the membrane fractions, samples were resuspended in the extraction/solubilization buffer (7M urea, 2 M thiourea, 2% C7BzO and 5 mM tributyl phosphine [TBP]). Matching fractions (F1**_wt_**+F1**_CIP_** and F2**_wt_**+F2**_CIP_**) were mixed at concentration ratios of 1∶1 (**^12^**C-light: **^13^**C-heavy) and subjected to separation by SDS-PAGE as a single sample. The mixed fractions are denominated pooled F1 and pooled F2, respectively. After separation by SDS-PAGE, the pooled F1 and F2 membrane fractions complete lanes were sliced into small gel bands (Fig. 2B). Each band was in-gel digested as previously described [84]. In short, gel bands were first destained by two consecutive washes in 150 µl buffer containing 200 mM ammonium bicarbonate in 50% (v/v) acetonitrile in water for 30 min at 30°C. After drying the gel pieces in a Speedvac (Thermo Savant, Holbrook, NY), 10 µl trypsin solution (0.002 µg/µl in a 50 mM ammonium bicarbonate buffer solution) was added and allowed to be absorbed by the gel for 40 min on ice. Gel bands were completely immersed by adding additional buffer solution and incubated at 37°C. After overnight digestion, tryptic peptides were extracted by 2 consecutive washes in 50 µl buffer (60% (v/v) acetonitrile, 0.1% (v/v) formic acid in water). Pooled peptide extractions were subsequently dried in a speedvac, dissolved in 15 µl 2% (v/v) acetonitrile, 0.1% (v/v) formic acid in water and stored at -20°C for further analysis.

### Liquid Chromatography Electrospray Ionization Fourier Transform Tandem Mass Spectrometry (LC-ESI-FT-MS/MS)

Tryptic peptides from each gel band were individually analyzed via a fully automated LC-MS/MS setup. Peptides were first separated on an Agilent 1200 chromatographic system (Agilent, Santa Clara, CA) and on-line measured on a LTQ-FT Ultra mass spectrometer (Thermo Fisher Scientific, Waltham, MA). Peptides in 5 µl solution were initially concentrated and desalted on a Zorbax 300SB-C18 trapping column, 5 mm×0.3 mm, at a 4 µl/min flow rate using a 2% (v/v) acetonitrile, 0.1% formic acid in water buffer. After valve switching, eluted peptides were separated on a Zorbax 300SB-C18 analytical column, 150 mm×75 µm (Agilent), by a 50 min linear gradient ranging from 2% (v/v) to 80% (v/v) acetonitrile, 0.1% formic acid in water at a 250 nl/min flow rate. Eluting peptides were analyzed on-line in the mass spectrometer via nano-ESI. Protein digests from fraction F2 were measured using the LTQ-FT Ultra nano-ESI source and applying a 1.1 kV voltage on PicoTip emitters (New Objective, Woburn, MA). For fraction F1 digests, LC-effluent was directly coupled to a Triversa NanoMate ESI source (Advion, Ithaca, NY), working in nano-LC mode and equipped with D-chips whereon a 1.55 kV voltage was supplied. The LTQ-FT Ultra mass spectrometer was tuned and calibrated with caffeine, MRFA (met-arg-phe-ala peptide) and UltraMark before measurement. The Fourier-transform ion cyclotron resonance (FT-ICR) mass analyzer acquired MS scans at 100,000 resolution during the LC separation. The 3 most intense precursor peptides for each MS scan were automatically selected and fragmented by the LTQ ion trap mass analyzer, and after 2 occurrences, precursor masses were excluded for 90 sec.

### Data Processing and Analysis

Raw LC-MS/MS data were analyzed with the Sequest database-searching algorithm [85] implemented in the Bioworks v3.3.1 software (Thermo Fisher Scientific). MS/MS spectra were searched against the mouse uniprot protein database concatenated with a shuffled decoy database generated by the Decoy Database Builder software tool [86] Propionamide on cysteines (+71.037114) and SILAC labels in arginine and lysine (+6.020129) were allowed as variable modifications. Mass tolerances were set to 10 ppm and 0.5 amu for peptide mass and fragment mass respectively. Two miscleavages and only b- and y-ions were considered. Raw LC-MS/MS data and Sequest result files were loaded into the TPP software v3.4 (Seattle Proteome Center [87]). Peptide identifications were validated with PeptideProphet filtering out peptide hits with probabilities below 0.70. Retained peptide identifications were subsequently quantified via the ASAPRatio tool using a fixed scan range for Light and Heavy labeled peptides and a 6.02013 Da label mass for both arginine and lysine. Mass tolerance was set to 0.01 Da. For each individual LC-run, peptide identifications and quantifications were grouped via the ProteinProphet tool and afterwards exported as excel files for further processing. All peptide and protein L/H ratios were manually controlled and if necessary adjusted. Manually validated protein ratios for each individual LC-run were grouped for redundant protein identifications via in-house written VBA (Visual Basic Scripting Edition)-scripts. Only proteins with a probability of 0.95 or higher were retained in the script. Final protein abundance ratios were subsequently calculated by averaging the logarithm base 2 protein ratios of an individual protein present in multiple gel bands. The final proteins ratios were normalized towards the median and their p-values calculated based on Z-statistics as previously described [88]. Unambiguously identified and quantified proteins from pooled fractions F1 and F2 were submitted to the online available DAVID Bioinformatics tool [89] and analyzed via their respectively GO terms for cellular localization and biological process. All MS data are available on the EBI’s PRIDE mass spectral depository (accession numbers 17116–17117). All samples were run in duplicate, as previously done by other groups using the SILAC technique [8], [90]. A main advantage of this approach resides indeed in the fact that it allows a relative quantification of proteins in a single sample obtained by mixing material collected from two different experimental conditions (in our case, WT and CIP-R cells), reducing the need of replicated samples. Moreover, quantitation is based on ratio’s obtained from different peptides per protein for which we used stringent conditions to accept a ratio as significant.

## Supporting Information

Figure S1 **Amino acid sequence of the cathepsin B precursor (P10605).** Arrows indicate the cleavage sites for the proteolytically activation of the protein precursor : Signal peptide (1–17), Propeptide (18–79), Cathepsin B (80–333), Light chain (80–126), Heavy chain (129–333) and Propeptide (334–339). Underlined sequences represent tryptic peptides identified specifically in gel bands 53–58 as shown in figure 2. Sequences in bold represent tryptic peptides identified specifically in gel bands 64 and 65. Not all tryptic peptides depicted here are identified in all mentioned gel bands, although their respectively precursors are present in the LC-MS data; except for three precursors specific for the upper gel bands which were absent in the lower bands (sequences in italic), demonstrating the appearance of the intact precursor in the upper gel region and the processed cathepsin B in the lower region.
(TIF)

Table S1 List of the 900 proteins identified (with at least 3 unique peptides using a 0.95 significancy threshold [P-value]). (651 proteins in fraction F1, 735 proteins in fraction F2).
(XLS)

Table S2 List of proteins represented by only 1 or 2 peptides using a 0.95 significancy threshold [P-value]). (386 proteins in fraction F1, 381 proteins in fraction F2).
(XLS)

Table S3 Distribution of cathepsin B (P10605) within the SDS-gel lane of pooled sample F1 and the respectively protein abundance ratios as calculated by ASAPRatio for each individual gel band.
(DOC)
