# Supplementary material for: Analysis of the Membrane Proteome of Ciprofloxacin-Resistant Macrophages by Stable Isotope Labeling with Amino Acids in Cell Culture (SILAC)
Source: PLoS One. 2013 Mar 7;8(3):e58285. doi: 10.1371/journal.pone.0058285 (PMC3591400; doi:10.1371/journal.pone.0058285)
Supplement: Table S3 — Distribution of cathepsin B (P10605) within the SDS-gel lane of pooled sample F1 and the respectively protein abundance ratios as calculated by ASAPRatio for each individual gel band. (DOC) [file pone.0058285.s004.doc]

**Table S3 :** Distribution of cathepsin B (P10605) within the SDS-gel lane of pooled sample F1 and the respectively protein abundance ratios as calculated by ASAPRatio for each individual gel band

| **band no. ^a^** | **# peptides ^b^** | **log_2_(L/H) ^c^** |
| --- | --- | --- |
| 53 | 4 | 1.43 |
| 54 | 9 | 1.40 |
| 55 | 11 | 1.32 |
| 56 | 14 | 1.06 |
| 58 | 3 | 0.90 |
| Average |  | **1.22** |
| (P-value) |  | **(0.022)** |
| 64 | 10 | 0.31 |
| 65 | 10 | -0.09 |
| Average |  | **0.11** |
| (P-value) |  | **(0.837)** |
| TOTAL |  |  |
| Average |  | **0.91** |
| (P-value) |  | **(0.091)** |

**^a^** ID number of gel slice from Figure 2

**^b^** number of identified unique tryptic peptides

**^c^** normalized protein ratio in log base 2 scale
